# VALIDATE-PERIANAL: an international real-world multi-centre exploratory validation of the TOpClass definition of a radiologically healed fistula in perianal fistulising Crohn’s disease

**DOI:** 10.1186/s13244-026-02260-1

**Published:** 2026-04-07

**Authors:** Easan Anand, Thirza J. van Croonenburg, Sami Samaan, Phil Tozer, Ailsa Hart, Parakkal Deepak, David H. Ballard, Jaap Stoker, Phillip Lung

**Affiliations:** 1 St Mark’s, The National Bowel Hospital, London, UK; 2 Department of Surgery & Cancer, Imperial College London, London, UK; 3 Department of Radiology and Nuclear Medicine, Amsterdam University Medical Centre, University of Amsterdam, Amsterdam, The Netherlands; 4 Amsterdam Gastroenterology Endocrinology Metabolism, Amsterdam, The Netherlands; 5 Washington University School of Medicine in St Louis, St Louis, MO USA; 6 Department of Metabolism, Digestion & Reproduction, Imperial College London, London, UK

**Keywords:** Fistula, Crohn’s disease (perianal), Colorectal imaging, MRI, Education

## Abstract

**Objectives:**

Radiological healing on MRI is the ultimate therapeutic goal in Class 2a perianal fistulising Crohn’s disease (pfCD). The TOpClass consortium recently defined radiological healing (TOpClass-RH) by the absence of T2 hyperintensity, a completely fibrotic fistula tract, and the absence of contrast enhancement when used. This study aimed to validate TOpClass-RH in real-world clinical practice.

**Materials and methods:**

VALIDATE-PERIANAL was a retrospective, multi-centre international study. Patients with pfCD with a baseline MRI scan showing active disease and follow-up MRI evidence of radiological fistula healing between 2021 and 2023 were identified. Paired scans were independently reviewed by three gastrointestinal radiologists using TOpClass-RH criteria, with consensus adjudication. A minimum of 12 months of clinical follow-up was required. The primary outcome was sustained clinical remission; secondary outcomes included inter-rater reliability (Cohen’s kappa) and rates of proctectomy and stoma formation.

**Results:**

Of 977 patients screened, 40 with pfCD met the inclusion criteria;14/40 (35%) fulfilled TOpClass-RH criteria. Sustained clinical remission was achieved in 93% of TOpClass-RH patients. Clinical recurrence occurred in 1/14 (7%) of TOpClass-RH patients versus 8/26 (30.8%) in the non-RH group (RR 4.3 [0.60–31.0], *p* = 0.12), with a median follow-up of 28 months. Inter-rater reliability was excellent (κ 0.89–0.95). There was a trend toward lower rates of proctectomy and stoma formation in the TOpClass-RH group.

**Conclusion:**

TOpClass-RH was associated with sustained clinical remission, although the study was underpowered to detect statistically significant differences. The definition demonstrated excellent inter-rater reliability, supporting TOpClass-RH as a clinically meaningful radiological endpoint for trials and diagnostic stratification in pfCD. Larger prospective studies are required.

**Critical relevance statement:**

This study validates the TOpClass criteria for defining a radiologically healed fistula in perianal Crohn’s disease, providing radiologists with practical pearls, pitfalls, and illustrative figures that advance clinical radiology practice and research application in MRI interpretation and trial standardisation.

**Key Points:**

- The TOpClass definition provides an internationally agreed and standardised MRI-based criteria for identifying a radiologically healed fistula in perianal Crohn’s disease.
- 93% of patients who met the TOpClass criteria for a radiologically healed fistula achieved sustained clinical remission, with excellent inter-rater reliability.
- The TOpClass criteria offer a potential benchmark for clinical trials, where a radiologically healed fistula represents the aspirational gold standard, associated with long-term remission in perianal fistulising Crohn’s disease.

**Graphical Abstract:**

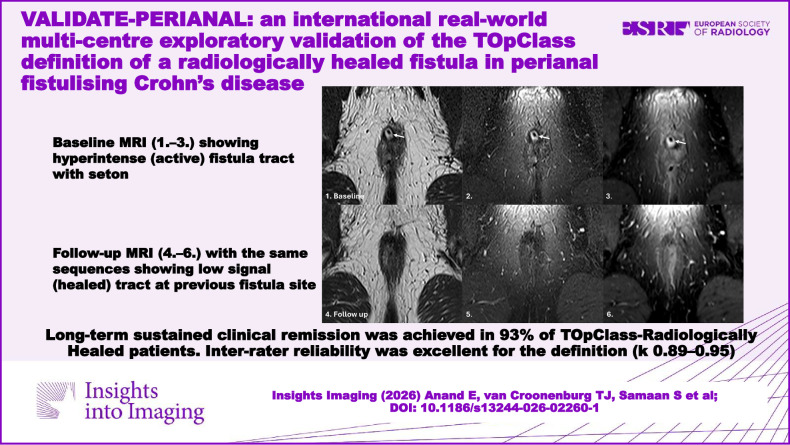

## Introduction

Perianal fistulising Crohn’s disease (pfCD) affects 20% of patients with Crohn’s disease [1] and is associated with substantial morbidity and impaired quality of life [2, 3]. It is characterised by treatment-refractory perianal fistulae that are notoriously difficult to treat with frequent recurrence despite medical and surgical interventions [4, 5].

MRI is the reference standard for assessing treatment response in pfCD as recommended by European Society guidelines [6]. Complete fistula healing on MRI remains the preferred therapeutic endpoint in class 2a patients [7]. The PISA II study demonstrated a correlation between radiological healing and sustained fistula closure, as well as a reduction in clinical recurrences, when compared to clinical healing alone [8, 9]. Radiological remission typically lags behind clinical remission but is more robust and sustained [10] with increasingly fibrotic tracts on MRI predictive of long-lasting clinical remission [11]. Despite over half of current trials in pfCD using radiological endpoints as either a primary or combined endpoint [12], there is currently no universally accepted definition of a radiologically healed fistula [13, 14].

To address this inconsistency in research standards, the TOpClass consortium of an international panel of experts in pfCD [7] has established consensus criteria for radiologically healed fistulae based on strict MRI features (Supplementary Material) [15]. The TOpClass completely radiologically healed (RH) fistula is defined by the complete absence of T2-weighted hyperintense signal in the fistula tract, a completely fibrotic tract, and the absence of contrast enhancement on post-contrast T1-weighted images when used [15]. This represents a paradigm shift from prior clinical trials like ADMIRE-CD, which defined radiological remission primarily by the absence of large MRI collections [16] and differs from PISA-II by not requiring IV contrast [8]. However, key uncertainties around clinical utility, reliability, feasibility, and generalisability of the TOpClass definition across varied imaging protocols require validation outside of trial settings. Such protocols include fistula MRI with T2- and T1-weighted sequences (with and without fat suppression), multiplanar acquisitions (sagittal, coronal, and axial), and the use of contrast enhancement.

To address these gaps, this exploratory study aimed to validate the international consensus definition of a radiologically healed fistula by applying it to real-world MRI datasets from multiple tertiary centres. Radiological healing outcomes were compared with established clinical endpoints, including fistula closure and recurrence, to evaluate prognostic value. Using the COSMIN framework, key measurement properties such as validity, reliability, and responsiveness were rigorously assessed [17, 18].

## Materials and methods

### Aims and objectives

The primary objective was to validate the TOpClass-Radiologically Healed (RH) definition in a real-world clinical setting, assessing its ability to predict sustained clinical remission. Secondary aims include determining whether achieving TOpClass-RH was reliable and accurately predicts long-term clinical outcomes compared to those who do not achieve these criteria.

### Study design and setting

VALIDATE-PERIANAL was a retrospective observational cohort study conducted at three tertiary referral centres with expertise in pfCD: Washington University School of Medicine in St. Louis (St. Louis, MO, USA), Amsterdam UMC (Amsterdam, The Netherlands) and St Mark’s the National Bowel Hospital (London, UK) using real-world MRI and clinical data. Patients were identified through institutional databases and radiology information systems over a 3-year period between January 2021 and December 2023. A minimum of 12 months of clinical follow-up was required from the date the MRI scan was reported as radiologically healed to ensure adequate assessment of durable healing. MRI scans performed prior to this period were excluded to ensure the inclusion of high-quality, consistent local protocol studies.

### Participants

Eligible participants were adults (≥ 18 years) with a diagnosis of pfCD in whom radiological healing or a completely radiologically healed fistula was reported by abdominal radiologists who routinely interpret perianal fistula protocol MRs. Inclusion criteria were the availability of a baseline pelvic MRI, at least one follow-up MRI after intervention reporting a healed fistula, and a minimum of 12 months of clinical follow-up from the date the MRI scan was reported as healed. For retrospective patients not previously enrolled in prospective registries, local ethical approval and patient re-consent were obtained where required.

### Data sources and cohort identification

Radiology information systems were queried using keywords (relevant Dutch terms in brackets), including “Healed fistula” (*Genezen fistel*), “Healed” (*Genezen*), “Fibrosis” (*Fibrose*), “Scarred” (*Litteken/Littekenweefsel*), “Resolved”, and “No longer visible” (*Niet meer zichtbaar*) to identify patients with potential radiologically healed fistulae (Crohn’s and non-Crohn’s fistulae were not distinguished during the search). MRI and clinical records were reviewed to confirm eligible pfCD patients. Data were extracted using a standardised collection template across all sites.

### MRI image acquisition

MRI was performed using 1.5-T (Siemens Avanto Fit) or 3.0-T (Philips Ingenia) scanners with a torso phased-array surface coil to obtain standard axial, coronal, and sagittal T2-weighted SPectral Attenuated Inversion Recovery sequences (TR 8000 ms; TE 96 ms; slice thickness 4 mm; gap 0.2 mm; FOV 240 mm). The complete protocol is included in Appendix C.

### MRI review and radiological endpoints

MRI scans were reviewed centrally on local PACS and via PACSBin (Orion Medical Technologies, LLC), enabling remote participation by three gastrointestinal radiologists (P.L., D.B., J.S.) with 5–30 years’ experience in MRI fistula reporting and active research in perianal Crohn’s disease (pfCD). Scan quality was graded as adequate or inadequate; scans lacking a baseline active MRI, insufficient field of view, or absent T2 fat-saturated sequences were excluded. Radiologists were trained using example cases and instructional materials to ensure consistency, followed by a consensus discussion to standardise interpretation of the TOpClass criteria. The TOpClass system accounts for multiple tracts: if one heals or improves while another worsens or a new tract develops, the overall classification is considered ‘not improved’, reflecting composite disease status rather than isolated tract changes.

Anonymised paired baseline and follow-up ‘healed’ scans were independently scored using the binary TOpClass-radiologically healed (RH) or radiological improvement (RI) criteria [15], followed by consensus adjudication when at least two of three readers agreed. Reviewers were blinded to patient identity and clinical outcomes. Inter-rater reliability was assessed using Cohen’s kappa and percentage agreement. The panel maintained detailed notes during review to document diagnostic uncertainty, inter-observer variation, and interpretative nuances, which informed iterative refinement of the TOpClass-RH criteria.

### Clinical outcomes

Clinical response and remission were assessed using a modified fistula drainage assessment (FDA) [19], applying gentle pressure over the external opening to evaluate discharge. For incomplete retrospective notes, fistulae were considered active unless stated otherwise. Clinical response was defined as a reduction in the number of draining fistulae, and remission as the complete absence of drainage on repeated examinations over 6 months. Patients with ongoing discharge or requiring further medical or surgical escalation were classified as non-responders. Outcomes were recorded at baseline (active disease) and at the most recent clinical follow-up.

### Sample size

This was an initial validation study: no formal power calculation was performed, and no predefined sample size was required. A 3-year search across three tertiary referral centres was anticipated to provide a sufficient number of cases for validation, with inclusion dependent on meeting the predefined eligibility criteria.

### Statistical analysis

Analyses were performed in IBM SPSS Statistics (v29). Baseline demographics and MRI characteristics were summarised descriptively; categorical variables are reported as percentages and continuous variables as mean (SD) or median (IQR). Inter-rater agreement was evaluated using Cohen’s kappa.

### Ethics and data governance

This study utilised data from the Perianal Fistula Database (REC reference: 19/NW/0447; IRAS project ID: 259750). Ethical approval was obtained from institutional review boards at all participating sites. Data were pseudo-anonymised in compliance with GDPR and local data protection standards. Re-consent for retrospective cases was obtained where required.

## Results

**Fig. 1:**
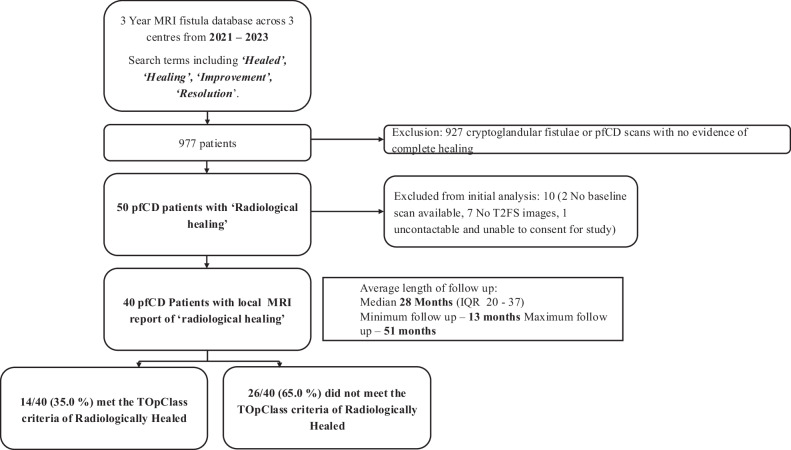
Study flowchart

A comprehensive radiology information systems review of MRI fistula scans across three centres identified 977 ‘healed’ patients over 3 years (Fig. 1). Most were excluded as they were cryptoglandular fistulae or did not report healed pfCD fistulae, leaving 50 eligible patients. Ten were further excluded due to absent baseline active scans (*n* = 2), missing T2 fat-saturated sequences (*n* = 7), or inability to consent (*n* = 1), yielding a final cohort of 40 patients (mean age 39 ± 11 years; mean BMI 26 ± 6.1 kg/m²; 57.5% female) (Table 1).

**Table 1:** Patient demographics

Variable	Value (SD or %)
Age (years)	39 (11.2)
BMI (kg/m²)	26.2 (6.1)
Sex, *n* (%):	
Female	23 (57.5%)
Male	17 (42.5%)
Ethnicity, *n* (%):	
White	31 (77.5%)
Asian Indian	3 (7.5%)
Black	3 (7.5%)
Asian other	2 (5.0%)
American Indian	1 (2.5%)
Smoking status, *n* (%):	
Non-smoker	34 (85%)
Smoker	3 (7.5%)
Ex-smoker	3 (7.5%)

### Patient demographics and fistula characteristics

Median Crohn’s disease duration was 10 years (IQR 5–16; Table 2) and perianal disease 4 years (IQR 2–9), with median follow-up after a ‘healed’ scan of 28 months (IQR 20–37; range 13–51). Disease was most commonly ileocolonic (L3, 40.0%) or colonic (L2, 35.0%), with the majority classified as pfCD class 2a [7, 20] at baseline (75.0%). Fistulae were mainly transsphincteric (55.0%) or intersphincteric (42.5%), and 77.5% were complex per AGA criteria [21]. Most patients had a single external (60.0%) and internal (75.0%) opening; horseshoe abscesses (15.0%), supralevator extension (7.5%), and abscess cavities (12.5%) were less frequent. Treatment persistence (i.e., continuation of the same therapy) was common, mostly infliximab, with switches primarily from anti-TNFs or immunomodulators to ustekinumab, risankizumab, or upadacitinib. Three patients remained untreated, four discontinued baseline therapy, 16 continued the same therapy, and 17 switched (Table 3). Treatment trajectories were similar between TOpClass-RH and non-TOpClass-RH fistulae.

**Table 2:** Disease and MRI fistula characteristics

Variable	Value
Crohn’s disease duration (years)	Median 10 (IQR 5–16)
Perianal CD duration (years)	Median 4 (IQR 2–8)
Montreal A classification [20], *n* (%)	
A1 (≤ 16 years)	8 (19.5%)
A2 (> 16–40 years)	32 (80.5%)
Montreal B classification, *n* (%)	
B1 (inflammatory)	20 (50.0%)
B2 (stricturing)	11 (27.5%)
B3 (penetrating)	9 (22.5%)
Montreal L classification, *n* (%)	
L1 (terminal ileum)	10 (25.0%)
L2 (colon)	14 (35.0%)
L3 (ileocolonic)	16 (40.0%)
pfCD class [7] at baseline, *n* (%)	
1. Minimal/asymptomatic	3 (7.5%)
2a. Amenable for repair	30 (75%)
2b. Symptom control only	4 (10.0%)
2c-i. Rapid progression (stoma)	1 (2.5%)
2c-ii. Slow progression (stoma)	1 (2.5%)
3. Proctectomy required	1 (2.5%)
Parks classification, *n* (%)	
Intersphincteric	17 (42.5%)
Transsphincteric	22 (55.0%)
Suprasphincteric	1 (2.5%)
AGA fistula type [21], *n* (%)	
Simple	9 (22.5%)
Complex	31 (77.5%)
Number of external openings, *n* (%)	
0	1 (2.5%)
1	24 (60%)
2	12 (30%)
3	3 (7.5%)
Number of internal openings, *n* (%)	
1	31 (77.5%)
2	8 (20%)
3	1 (2.5%)
Horseshoe abscess, *n* (%)	
Absent	34 (85%)
Present	6 (15%)
Supralevator extension, *n* (%)	
Absent	37 (92.5%)
Present	3 (7.5%)
Abscess cavity present, *n* (%)	
No	35 (87.5%)
Yes	5 (12.5%)

**Table 3:** Crohn’s disease treatment baseline

Previous CD-luminal surgery at baseline	*N* (%)
No major luminal surgery	29 (72.5%)
Right hemicolectomy	7 (17.5%)
Subtotal colectomy	1 (2.5%)
Stricturoplasty	2 (5.0%)
Small bowel resection	1 (2.5%)
Previous perianal fistula repair attempts	*N* (%)
No fistula repair attempted	27 (67.5%)
Lay open	6 (15.0%)
LIFT	2 (5.0%)
MSC	2 (5.0%)
FiLac	2 (5.0%)
Baseline Crohn’s medication	*N* (%)
Infliximab	19 (47.5%)
Azathioprine	4 (10%)
Vedolizumab	5 (12.5%)
Adalimumab	2 (5%)
Ustekinumab	3 (7.5%)
Methotrexate	1 (2.5%)
Certolizumab	1 (2.5%)
Risakinuzumab	1 (2.5%)
Corticosteroids	1 (2.5%)
No medication	3 (7.5%)

### Primary outcome

**Fig. 2:**
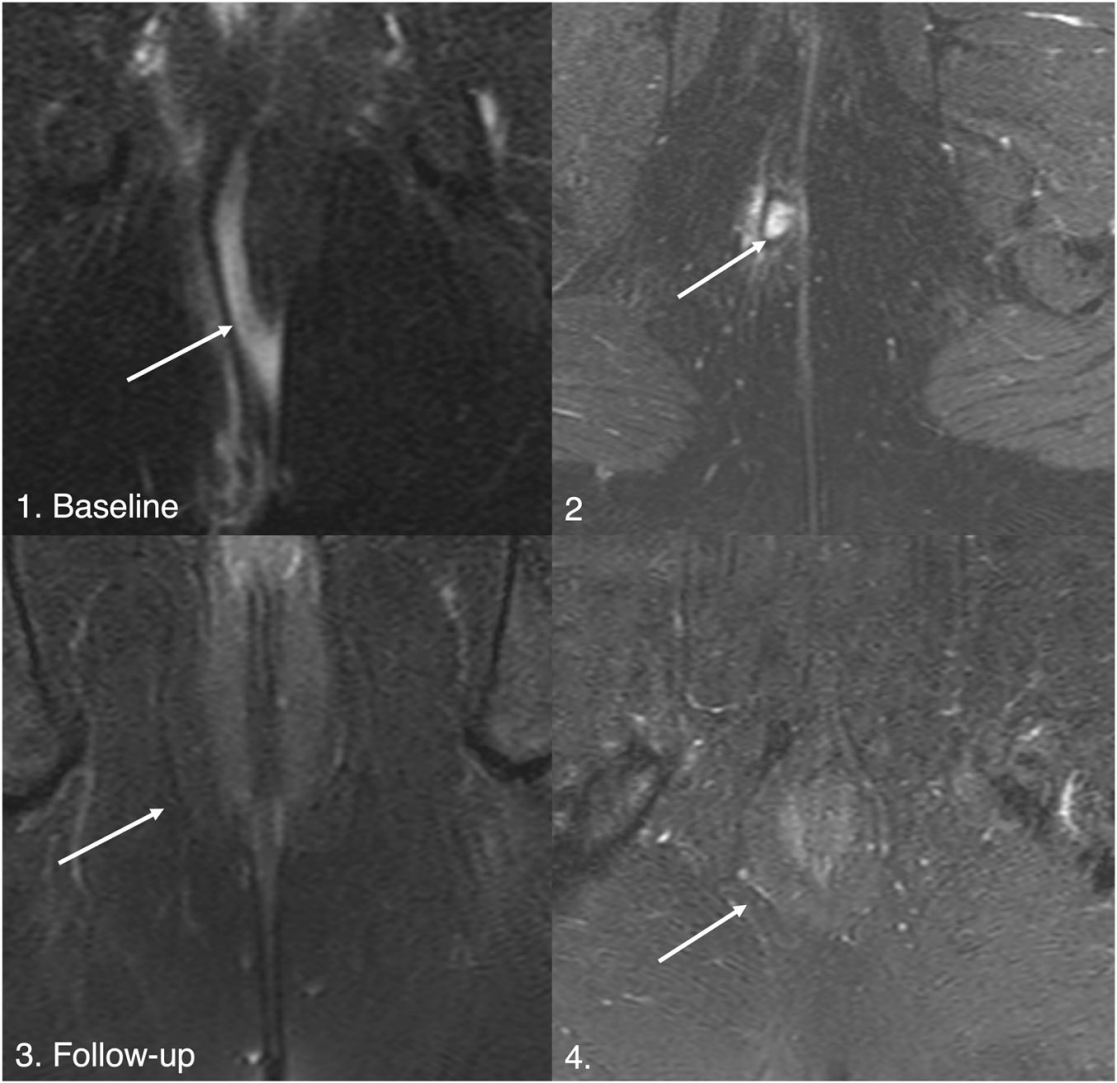
MRI non-contrast T2-weighted coronal and axial fat-saturated sequences illustrating TOpClass-radiologically healed fistula with sustained clinical remission. From top to bottom, left to right: Baseline MRI. **1** T2-weighted fat-saturated SPAIR coronal and (**2**) axial sequences depict an active transsphincteric fistula tract. Follow-up MRI. **3** Coronal and (**4**) axial sequences depict a TOpClass-radiologically healed fistula characterised by a complete absence of T2 hyperintensity and a completely fibrotic fistula tract

**Fig. 3:**
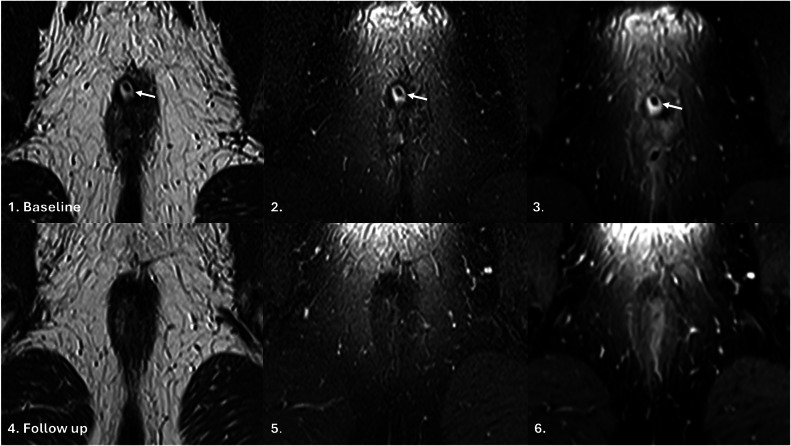
MRI axial oblique T2-weighted sequence, Fat-saturated T2-weighted sequence and post-contrast fat-saturated T1-weighted sequences illustrating TOpClass-radiologically healed fistula with sustained clinical remission. From top to bottom, left to right: Baseline MRI. **1** Axial oblique T2-weighted, (**2**) fat-saturated T2-weighted and (**3**) post-contrast fat-saturated T1-weighted images show a hyperintense tract (arrow) with a seton, consistent with ongoing fistula activity. Follow-up MRI (**4**–**6**) shows a hypointense tract at the previous fistula site on all three axial oblique sequences, consistent with fibrosis and absence of active inflammation, therefore, a healed fistula

**Fig. 4:**
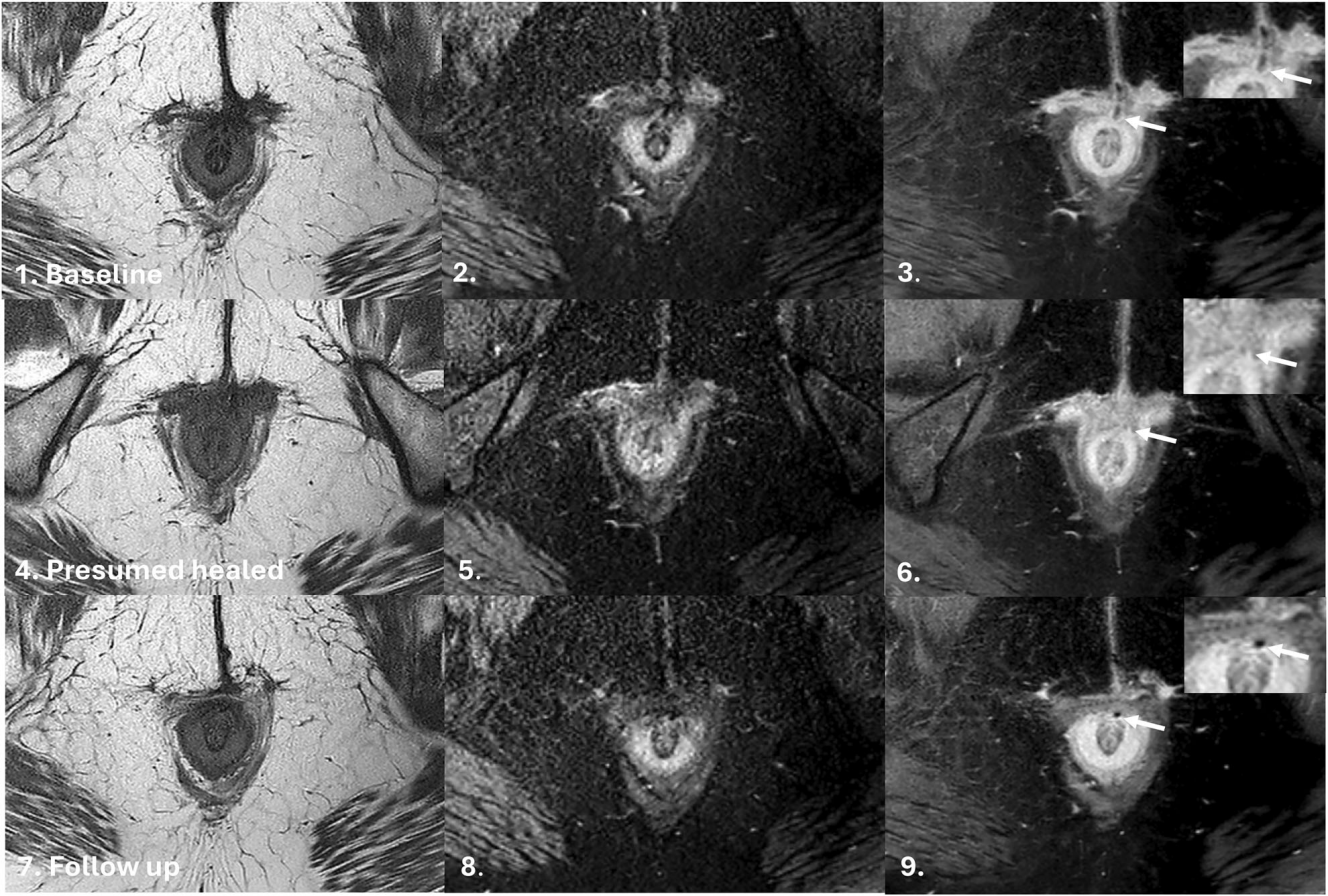
TOpClass-radiologically healed fistula with clinical recurrence. From top to bottom, left to right: Baseline MRI. **1** Axial oblique T2-weighted, (**2**) fat-saturated T2-weighted and (**3**) post-contrast fat-saturated T1-weighted images show subtle hyperintensity (arrow) with a central hypointense seton, consistent with ongoing fistula activity. Presumed healed MRI (**4**–**6**) shows a hypointense tract at the previous fistula site on all three axial oblique sequences, consistent with fibrosis and absence of overt active inflammation, meeting criteria for radiological healing. Follow-up MRI (**7**–**9**) (6 months later) demonstrates re-emergence of a hyperintense tract at the same site (arrow), consistent with recurrent fistula activity

Of 40 patients, 14 (35.0%) met TOpClass-RH criteria, while 26 (65.0%) did not, despite being reported as healed locally. Over a median follow-up of 28 months, 93% (13/14) of TOpClass-RH patients had sustained clinical remission versus 69.2% (18/26) of non-TOpClass-RH patients. An example of scans meeting TOpClass-RH criteria is shown in Figs. 2 and 3. Recurrence was observed in 8/26 (30.8%) non-TOpClass-RH versus 1/14 (7.0%) TOpClass-RH patients, corresponding to a relative risk of 4.3 (95% CI 0.60–31.0), indicating a trend towards a fourfold higher recurrence among non-TOpClass-RH patients, though this was not statistically significant (Fisher’s exact *p* = 0.12). The single TOpClass-RH recurrence corresponded to a subtle hyperintense signal which can be seen in the intersphincteric space (Fig. 4).

### Secondary outcomes

**Fig. 5:**
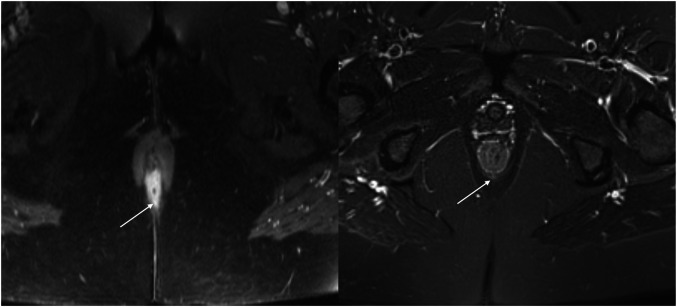
TOpClass–radiological improvement (not complete TOpClass-RH) with majority agreement for not healed. From left to right: Baseline MRI (left): Axial oblique fat-saturated T1-weighted image demonstrates an active fistula at the 6 o’clock position. Follow-up MRI (right): Axial oblique fat-saturated T2-weighted image demonstrates interval improvement but persistent subtle hyperintensity within the posterior anal sphincter (arrow), resulting in classification as not meeting TOpClass radiological healing criteria (2:1 reader agreement)

The TOpClass-RH and RI definitions were simple and binary, facilitating straightforward scoring. Inter-rater agreement amongst the three expert GI radiologists for TOpClass-RH was excellent (Cohen’s κ 0.89–0.95; total agreement 95.0–97.5%) (Table 4a). Two disagreements occurred: one 2:1 healed case with no clinical recurrence, and one 2:1 not-healed case with subtle residual posterior signal, later confirmed as a clinical transsphincteric fistula recurrence (Fig. 5). TOpClass-RI showed perfect agreement (κ = 1.00; 100% total agreement) across all readers (Table 4b), indicating complete concordance.

**Table 4:** Inter-rater reliability: **a** TOpClass-radiologically healed (RH) reliability; **b** TOpClass-radiological improvement (RI) reliability

a. Raters compared	Cohen’s κ (Kappa)	Inter-rater agreement (%)
Reviewer 1 vs Reviewer 2	0.95	97.5
Reviewer 1 vs Reviewer 3	0.89	95.0
Reviewer 2 vs Reviewer 3	0.94	97.5

b. Raters compared	Cohen’s κ	Inter-rater agreement (%)
Reviewer 1 vs Reviewer 2	1.00	100
Reviewer 1 vs Reviewer 3	1.00	100
Reviewer 2 vs Reviewer 3	1.00	100

Secondary outcomes, including hospitalisation, stoma formation, return to operating room, proctectomy, seton re-insertion, and antibiotic use, were less frequent in patients achieving TOpClass-RH (Appendix B). Relative risks trended lower for all events compared with non-TOpClass-RH patients, though no differences reached statistical significance. Notably, no TOpClass-RH patients required a defunctioning stoma, versus 15.4% in the non-healed group (RR 0.2, 95% CI 0.01–3.24, *p* = 0.28).

### Interpretation of radiologically healed fistula criteria

**Fig. 6:**
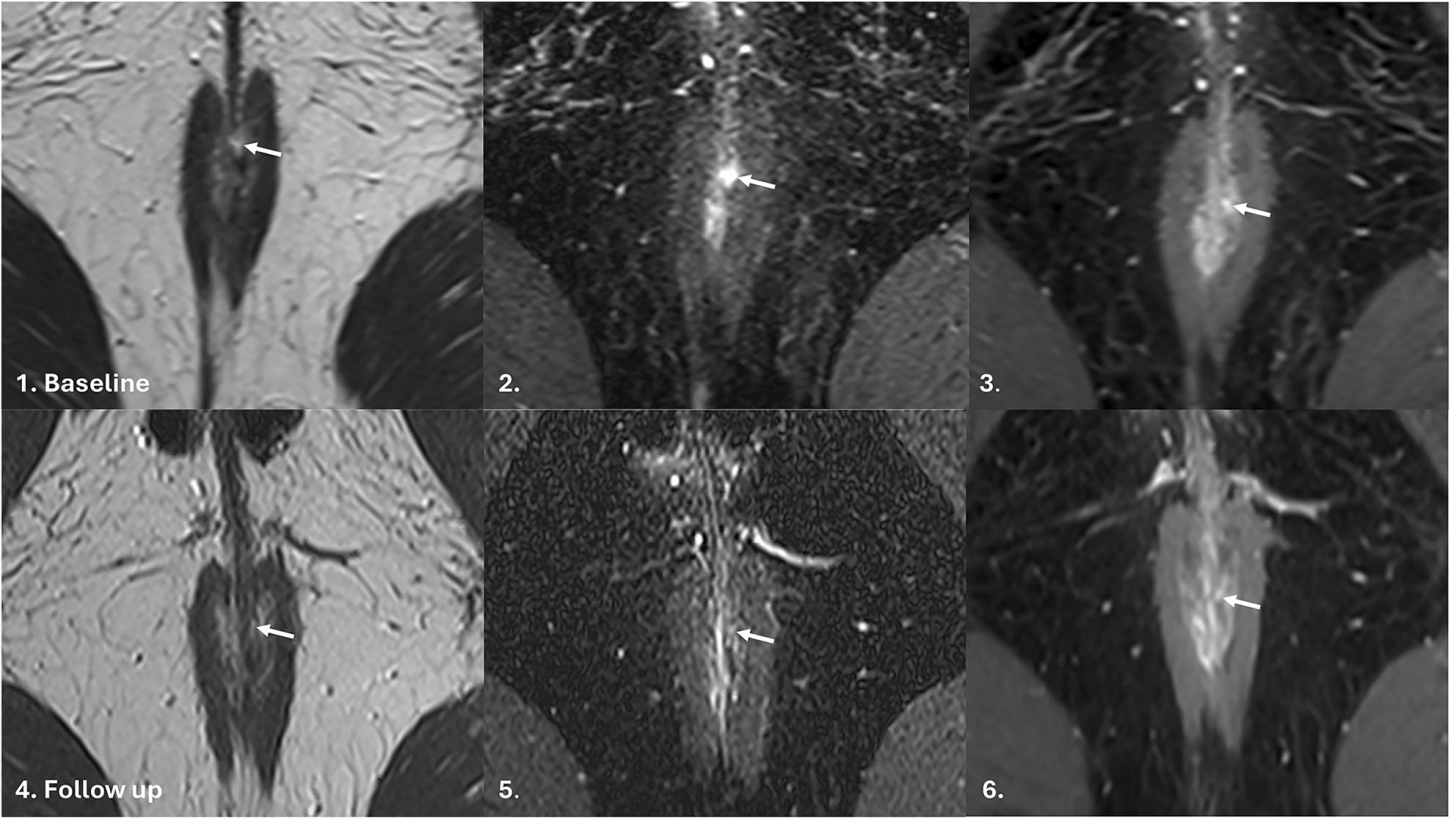
TOpClass-radiologically improved fistula with clinical recurrence. From top to bottom, left to right: Baseline MRI. **1** Axial oblique T2-weighted, (**2**) fat-saturated T2-weighted and (**3**) post-contrast fat-saturated T1-weighted images demonstrate a hyperintense tract (arrow), consistent with ongoing fistula activity. Follow-up MRI (**4**–**6**) (9 months later) demonstrates a focal area of residual hyperintensity (arrow), most conspicuous on the post-contrast fat-saturated T1-weighted image, consistent with radiological improvement but incomplete healing

Expert qualitative feedback identified four key themes relevant to the assessment of TOpClass-RH (Table 5). These included standardised imaging protocols and sequences, identification of anterior or anovaginal fistulae, confounding features and the need for longitudinal follow-up. The importance of subtle residual disease activity indicating potential disease recurrence is illustrated in Fig. 6.

**Table 5:** Radiologically healed fistula interpretation—“pearls and pitfalls”

Take-home messages	Key considerations
1. Key imaging protocols and sequences	Ensure consistent imaging protocols across centres (TOpClass recommendations [15]), including: • Fistula protocol MRI with appropriate field of view (FOV) • T2-weighted sequences (with and without fat suppression) • Multiplanar acquisitions (sagittal, coronal, and axial). Motion artefact and image degradation should be avoided—high-quality images are required for diagnostic certainty. While IV contrast is not a standard component of routine clinical fistula MRI in every institution, axial T1-weighted images with fat suppression after administration of a gadolinium-based contrast agent may be necessary to improve standardisation and diagnostic confidence, particularly when healing is uncertain, for example, anovaginal fistulae (AVF). Evidence from long-term follow-up of the PISA II data [9] suggests fat-saturated T1 post-contrast sequences are the most sensitive for identifying subtle residual signal intensity (and therefore incomplete healing and disease recurrence).
2. Anterior/anovaginal fistulae	• Anterior fistulae/AVF are difficult to identify, and IV contrast may be used to increase diagnostic certainty. • Where healing is uncertain, IV contrast and/or EUA (particularly for AVF cases) may assist in decision-making. • Residual signal or subtle enhancement should be treated with caution, as this may indicate partial healing.
3. Features to be aware of	Be aware of factors such as: • Any residual T2 signal or enhancement within the fistula tract (including wall) that can indicate a partially healed tract. In our expert opinion, a repeat MRI with IV contrast may be considered at 12 months. • A completely fibrotic tract with surrounding oedema* can still be considered a radiologically healed fistula. • T2 hyperintensity not due to active tract (e.g., vessel). • In a tract that would otherwise be considered radiologically healed, **high signal near internal openings may indicate partially healing or be due to distortion. ^*^ On MRI, oedema may mimic fistula activity as both appear hyperintense on T2-weighted sequences; however, oedema typically manifests as diffuse, ill-defined signal within adjacent fat without a discrete morphology or continuous course. ^**^ On MRI, residual inflammation at the internal opening may be suggested by focal high T2 signal with avid enhancement and ill-defined margins, while healed change appears low-to-intermediate on T2 with minimal enhancement and well-defined borders.
4. Need for longitudinal follow-up	• Radiological improvement does not always equate to durable remission. • Longitudinal clinical and radiological follow-up is essential for verifying true healing, particularly in borderline or uncertain cases.

## Discussion

The TOpClass definition of a radiologically healed fistula in pfCD is a clinically meaningful and reliable endpoint. Across three large expert centres, 14/40 (35%) MRI scans reported as healed fulfilled TOpClass-RH criteria. Of these, 93% achieved sustained clinical remission over a median 28-month follow-up, compared to 69% remission in those not meeting TOpClass-RH, although the study was underpowered in this exploratory validation study to detect a statistically significant difference between these 2 closely related groups. The TOpClass-RH criteria also demonstrated almost perfect inter-rater agreement with over 95% agreement for radiological healing among three experienced readers and κ values between 0.89 and 0.95. We have demonstrated the reliability of the consensus definitions, rendering them suitable for application in larger prospective studies and clinical trials.

These findings align with the multi-centre PISA II trial [22], which reported no clinical recurrences among patients with radiological healing using criteria similar to those applied here, in contrast to recurrences among those who achieved clinical closure but not radiological healing. In our study, patients not meeting TOpClass-RH trended towards higher recurrence risk (RR 4.3), though this was not statistically significant.

Any residual T2 signal or enhancement within the fistula tract (including wall) can indicate a partially healed tract. The single case of recurrence within the TOpClass-RH group (Fig. 4) provides an important educational insight for radiological assessment of perianal fistula healing. Retrospective review of this case demonstrated very subtle residual hyperintensity within the intersphincteric space on the presumed healed MRI, consistent with persistent low-grade inflammatory activity that was initially underappreciated. This highlights the critical importance of systematically scrutinising follow-up MRI for even minimal residual signal, particularly in anatomically complex regions such as the intersphincteric plane. Within the TOpClass-RH framework, any persisting hyperintensity or equivocal signal should be explicitly reported and classified as “not healed”. Adoption of this conservative approach is essential to reduce false-negative classification and to support safe clinical decision-making based on radiological healing.

There is an absence of evidence on distinguishing signal outside the fibrous fistula tract from active disease on MRI, and in light of this study’s findings, repeat MRI may be a cautious yet sensible approach. In this setting, one should consider including a fat-saturated T1 post-contrast sequence, as data from the PISA II trial [9] suggest that this sequence is the most sensitive for identifying subtle residual signal intensity. Findings from this retrospective study suggest that rigorous application of the TOpClass-RH definition can more reliably identify patients who will remain in sustained clinical remission than existing standards. By contrast, a subtle residual signal may be responsible for disease recurrence in the long-term. Similarly, PISA-II showed that clinical recurrences were noted in those with an almost completely fibrotic tract and only minimal MRI activity, although absolute numbers were not described in the paper [22].

Secondary clinical outcomes such as hospitalisation, proctectomy, switch in biologic therapy, seton re-insertion, and return to operating room were all consistently less frequent among patients who achieved TOpClass-RH, supporting its potential as a prognostic radiological biomarker with fewer adverse disease-related events, although the sample size was too low to detect a statistically significant difference.

To improve consistency in assessment, we have developed a TOpClass-RH interpretation guide with recommendations and examples of healed and improved fistulae (Figs. 2–6) based on consensus from expert GI radiologists, facilitating standardisation of MRI evaluation for fistula healing. We propose that the presence of even a minimal signal change should preclude classification as TOpClass-RH and instead be considered TOpClass-RI. This emphasises the importance of systematic comparison with prior MRI, as interval reduction or stability of subtle signal is more consistent with improvement, whereas new or increasing activity would suggest persistence or progression.

### Pearls and pitfalls of interpreting TOpClass-radiologically healed criteria

Discussion throughout the consensus reads illustrated the importance of standardised imaging protocols (e.g., T2 fat-saturated sequences, selective use of IV contrast), pragmatic consensus reading of paired scans to reflect real-world practice, careful identification of anovaginal fistulae, differentiating partial healing from complete healing, awareness of confounding features such as residual signal and oedema, and the need for longitudinal follow-up to confirm durable remission. Specifically, a completely fibrotic tract is only made up of scar tissue (i.e., no inflammation in a previously active tract). Subtle residual T2 signal is any trace of hyperintense signal within the fistula tract at (fat-saturated) T2-weighted sequences; minimal enhancement is the presence of any T1 hyperintensity in the fistula tract on post-contrast imaging; oedema manifests as diffuse ill-defined signal within adjacent fat without a discrete morphology or continuous course—all are features that should be treated with caution as they may indicate only partial or incomplete healing. These considerations aim to improve consistency, interpretive accuracy, and clinical relevance in both routine practice and research settings. Although IV contrast use in pelvic MRI for pfCD varies in practice, this study highlights its value in improving diagnostic confidence for radiological healing, especially in borderline cases or when T2 signal changes are subtle. Figure 6 illustrates the subtleties of persistent/residual disease activity (radiological improvement with clinical recurrence), which appears to be associated with clinical recurrence. However, until high-quality prospective studies provide further evidence, we should be cautious in defining complete TOpClass RH, and repeat MRI (with consideration of IV contrast) may be required to confirm this assessment.

We recognise that the TOpClass-RI category encompasses a broad spectrum of disease response. This lack of granularity may limit its clinical and research utility. Future work should therefore aim to refine this category, for example, through stratification using validated MRI indices such as MAGNIFI-CD [23] or quantitative measures such as percentage reduction in tract volume [24–26]. Such approaches could provide a more nuanced assessment of radiological response, enabling more consistent evaluation of treatment outcomes.

### Strengths

This study is strengthened by an extensive and systematic search of MRI scans from three expert tertiary referral centres, yielding a large potential dataset despite the absolute rarity of radiological healing. The inclusion of an average 28-month longitudinal follow-up following a ‘healed’ scan provides valuable insight into the long-term clinical relevance of radiological endpoints. The involvement of expert GI radiologists, each with between 5 and 30 years’ experience in pfCD, employing a consensus reading methodology ensured high-quality, reliable assessments, reflected in near-perfect inter-reader agreement for radiological improvement and healing. Furthermore, the refinement of the definition with key tips for interpretation and figure examples of TOpClass-RH and RI represents an important practical tool to promote standardised assessment in both clinical and research settings.

### Limitations

Despite these strengths, the study was limited by the small number of patients achieving TOpClass-RH across three centres, restricting statistical power. Although recurrence rates differed (7% vs 31%), the study was underpowered; 130–143 patients would be needed for 95% power. Trends towards higher recurrence in non-TOpClass-RH patients suggest radiological healing is a more robust endpoint than clinical closure. The identification of a cohort of ‘healed’ patients using keyword-based radiology information system searches is inherently prone to sampling bias, reflecting the absence of a universally accepted definition of radiological healing. This approach may exclude patients who genuinely meet strict TOpClass-RH criteria but were not explicitly labelled as healed in routine reports, potentially overestimating the prevalence of TOpClass-RH within clinical reporting (35%). Although the search strategy was as comprehensive as feasible across three tertiary centres, some cases of radiological healing were likely missed. This limitation may bias estimates of discordance between routine reporting and the TOpClass-RH standard and highlights the need for prospective studies including all patients with pfCD undergoing follow-up MRI, irrespective of reported findings. Heterogeneity in imaging protocols, including contrast use and MRI sequences, limited the inclusion of reference standards such as MAGNIFI-CD [23] and necessitated the exclusion of scans lacking T2 fat-saturated images. Future multi-centre studies should harmonise imaging practices and adhere to minimum reporting standards [27] to ensure adequate power and comparability, as highlighted in prior consensus exercises including ESGAR [6, 28] and TOpClass guidelines [15].

## Conclusions

The TOpClass-RH definition is a stringent, practical, and reliable radiological endpoint, which is associated with sustained clinical remission in 93% patients and > 95% inter-rater agreement amongst expert readers. However, complete radiological healing remains uncommon, even in expert centres. While TOpClass-RH is an aspirational gold standard, softer endpoints such as radiological improvement require further validation. Future, larger prospective multi-centre studies are needed to establish the external validity of the TOpClass criteria, evaluate impact on patient quality of life and refine imaging protocols. Standardised, reliable radiological endpoints will be essential for advancing therapeutic evaluation and improving outcomes in pfCD.

## Supplementary information

ELECTRONIC SUPPLEMENTARY MATERIAL

## Data Availability

The data underlying this study are derived from a multi-centre radiological validation project conducted across participating institutions. De-identified imaging datasets and associated clinical variables were collected in accordance with local ethical approvals and data-sharing agreements. Due to patient confidentiality and institutional policies, the raw data cannot be made publicly available.

## Abbreviation

pfCD: Perianal fistulising Crohn’s disease
TOpClass: Treatment Optimisation and Classification in perianal Crohn’s disease (A multidisciplinary consortium of international experts in perianal Crohn’s disease)
