# Supplementary material for: VALIDATE-PERIANAL: an international real-world multi-centre exploratory validation of the TOpClass definition of a radiologically healed fistula in perianal fistulising Crohn’s disease
Source: Insights Imaging. 2026 Apr 7;17:90. doi: 10.1186/s13244-026-02260-1 (PMC13057074; doi:10.1186/s13244-026-02260-1)
Supplement: Supplementary file 1 — ELECTRONIC SUPPLEMENTARY MATERIAL [file 13244_2026_2260_MOESM1_ESM.pdf]

VALIDATE-PERIANAL: An international real-world multi-centre exploratory validation of the TOpClass definition of a Radiologically Healed Fistula in perianal fistulising Crohn’s disease

ELECTRONIC SUPPLEMENTARY MATERIAL

Insights Imaging (2026) Anand E, van Croonenburg TJ, Samaan S, et al.

Appendix A: Definition of a radiologically improved fistula and a radiologically healed fistula on MRI in perianal Crohn's disease

**A radiologically improved fistula on MRI in perianal Crohn's disease can be defined by at least one essential criterion:**	**% Level of agreement (either strongly agree or somewhat agree)**
• An increasingly fibrotic fistula tract (compared to baseline)	97.6 % (76/78)
• An unequivocal reduction in:	
o Fistula tract T2-weighted hyperintensity compared to baseline study	93.6 % (73/78)
o Fistula tract diameter	92.3 % (72/78)
o Fistula tract length	83.4 % (65/78)
o Size or complete absence of previously present abscess	93.6 % (73/78)
• Where contrast is used:	
o An unequivocal reduction in fistula tract hyperintensity on contrast T1-weighted fat saturated images compared to baseline study	97.5 % (76/78)
**A radiologically healed fistula can be defined by the presence of the following essential criteria:**	
• Absence of T2-weighted hyperintense signal in fistula tract	94.9 % (74/78)
• Where contrast is used, the absence of contrast enhancement on post-contrast T1-weighted images	98.7 % (77/78)
• A completely fibrotic fistula tract *	97.5 % (76/78)

**Fibrosis (scar tissue) alone cannot always define healing as some fistulae leave no residual findings. A fistula tract can only be considered completely fibrotic if no inflammation is present.*

% Level of agreement shows the percentage of participants voting either strongly agree or somewhat agree after 2 rounds of online survey (this applies to all subsequent tables). At the final consensus meeting 80 % (16/20) attendees endorsed the definition of radiological improvement and 95 % (21/22) voted to confirm the definition of radiologically healed. Those selecting 'don't know' were not included in the overall result. All definitions and recommendations were circulated to and approved by all 84 members of the consensus panel.

Appendix B: Secondary Outcomes of interest

Comparison of Secondary Outcomes in Patients With TOpClass-RH vs. Not TOpClass -RH (n = 40)

Outcome	TOpClass-RH (n = 14)	Not TOpClass-RH (n = 26)	RR (95% CI)	p-value
**Hospitalisation**	1 (7.1%)	5 (19.2%)	0.37 (0.05 – 2.87)	.40
**Defunctioning stoma**	0 (0.0%)	4 (15.4%)	0.20 (0.01 – 3.24)	.28
**Return to operating room**	2 (14.2 %)	9 (34.6%)	0.41 (0.10 – 1.68)	.28
**Proctectomy**	1 (7.1 %)$^{+}$	3 (11.5%)	0.64 (0.07 – 5.73)	1.00
**Seton re-insertion**	1 (7.1 %)	5 (19.2%)	0.37 (0.05 – 2.87)	.40
**Fistula-related antibiotics**	1 (7.1 %)	5 (19.2%)	0.37 (0.04 – 2.87)	.40
**Stopped medication**	3 (21.0 %)	4 (15.0 %)	1.39 (0.36 – 5.37)	.63
**Stayed on same medication**	7 (50.0 %)	8 (31.0 %)	1.63 (0.75 – 3.54)	.23
**Switched medication**	4 (29.0 %)	14 (54.0 %)	0.53 (0.22 – 1.31)	.13

Note. RR = relative risk; CI = confidence interval. Fisher's exact test was used for p-values.

[*]A single patient accounted for hospitalisation, return to operating room, seton re-insertion, and fistula-related antibiotics; subtle T2 activity, not thought to be fistula tract-related) was noted, with clinical recurrence at 2 months requiring further intervention, and subsequent MSC therapy without achieving remission.

[+]One patient underwent proctectomy for intractable incontinence secondary to multiple EUAs and atrophic sphincter muscle on clinical examination and MRI, despite having a healed fistula on both radiological and long-term clinical follow-up.

Reasons for Return to Operating Room in TOpClass RH vs. Not TOpClass-RH

Group	Case	Reason for Return to Operating Room
**TOpClass-RH**	1	Clinical recurrence at 2 months requiring return to operating room, antibiotics, and seton insertion
	2	No evidence of fistula; underwent proctectomy for debilitating faecal incontinence and subacute obstructive symptoms
**Not TOpClass-RH**	1	Proctectomy for proctocolitis, incontinence, and rectal stricture (2024)
	2	EUA and lay open
	3	Parastomal abscess (unrelated to fistula)
	4	Mild perianal symptoms with seton in situ; future plans for removal

Group	Case	Reason for Return to Operating Room
	5	Perianal abscess requiring incision and drainage with antibiotics
	6	Total proctectomy for refractory disease with prolonged treatment gaps due to lack of insurance
	7	EUA and seton insertion ~2 years later for recurrent complex fistula
	8	Perianal fistula diagnosed on examination 2 years later
	9	Symptomatic perianal fistula diagnosed 2 years requiring EUA + Seton insertion

Reasons for Proctectomy in TOpClass RH vs. Not TOpClass-RH

Group	Case	Reason for Proctectomy
**TOpClass RH**	1	No clinical or radiological evidence of fistula; proctectomy performed for debilitating faecal incontinence and subacute obstructive symptoms
**Not TOpClass-RH**	1	Proctectomy for proctocolitis, incontinence, and rectal stricture
	2	Total proctectomy after refractory disease course with prolonged treatment gaps due to lack of insurance
	3	EUA and seton for recurrent complex fistula, later requiring proctectomy for intractable disease

Appendix C:

The imaging protocol included sagittal and coronal T2-weighted fast spin-echo sequences without fat suppression and axial T2-weighted sequences with and without fat suppression (TR ~2800 ms; TE ~70 ms; slice thickness 3 mm; gap 0.3 mm; FOV 300 mm). Post-contrast T1-weighted fat-suppressed images (Amsterdam UMC and Washington University in St Louis only) were acquired after intravenous gadolinium (0.1 mmol/kg Dotarem®, gadoterate meglumine; Guerbet, Villepinte, France), TR ~605 ms; TE ~9.6 ms; slice thickness 3 mm; gap 0.3 mm; FOV 300 mm). Coronal and axial planes were angulated parallel and perpendicular to the anal canal. Intravenous anti-peristaltic medication (20 mg scopolamine butylbromide, Buscopan; Boehringer Ingelheim Ltd., Ingelheim am Rhein, Germany) was administered before imaging. Digital Imaging and Communications in Medicine (DICOM) images were retrieved from the Picture Archiving and Communication System (PACS) and anonymised.
